# Design and Analysis of a Petri Net Model of the Von Hippel-Lindau (VHL) Tumor Suppressor Interaction Network

**DOI:** 10.1371/journal.pone.0096986

**Published:** 2014-06-02

**Authors:** Giovanni Minervini, Elisabetta Panizzoni, Manuel Giollo, Alessandro Masiero, Carlo Ferrari, Silvio C. E. Tosatto

**Affiliations:** 1 Dept. of Biomedical Sciences, University of Padua, Padua, Italy; 2 Dept. of Information Engineering, University of Padua, Padua, Italy; University of Rome Tor Vergata, Italy

## Abstract

Von Hippel-Lindau (VHL) syndrome is a hereditary condition predisposing to the development of different cancer forms, related to germline inactivation of the homonymous tumor suppressor pVHL. The best characterized function of pVHL is the ubiquitination dependent degradation of Hypoxia Inducible Factor (HIF) via the proteasome. It is also involved in several cellular pathways acting as a molecular hub and interacting with more than 200 different proteins. Molecular details of pVHL plasticity remain in large part unknown. Here, we present a novel manually curated Petri Net (PN) model of the main pVHL functional pathways. The model was built using functional information derived from the literature. It includes all major pVHL functions and is able to credibly reproduce VHL syndrome at the molecular level. The reliability of the PN model also allowed in silico knockout experiments, driven by previous model analysis. Interestingly, PN analysis suggests that the variability of different VHL manifestations is correlated with the concomitant inactivation of different metabolic pathways.

## Introduction

Pathological deregulation of cellular pathways often results in a family of complex and correlated diseases commonly termed cancer [1]. Cancer is a multi factorial disease where different causes contribute to its development. Several computational methods have been developed to explore the functional pathways involved in tumorigenesis. Some of them focus on differential gene expression between healthy and pathologic tissues [2]–[3], on protein-protein interaction network analysis [4]–[5] or on molecular dynamics simulations [6]. Other methods approach the disease through discretization of pathological components that result in tumor [7]. All of these approaches are very powerful when the variables related to the disease, although complex, are well known and studied. A multi-factorial disease can be approached by means of mathematical theory, building a theoretical model where cell components are connected with each other. In biology, several problems were dealt with network theory [8]–[9]. A network is a group of objects strongly inter-connected with each other (e.g. proteins and enzymes of a pathway or animals belonging to interacting populations). Their construction and subsequent simulation is made via mathematical analysis of the connections between nodes found in the system and their time-dependent behavior [10]. A biological network is generally composed of proteins, nucleic acids and cofactors connected by biological reactions such as protein complex formation or enzyme activity regulation [10]. Von Hippel-Lindau syndrome (VHL) [11] is a good study case to test the network theory applied to cancer due to the similar medical history and pathological phenotype that patients share. While hereditary cancers represent only a small part of all human tumors, their investigation represents a challenge to understand the pathway leading to tumor formation. In 2010, Heiner et al. first approached VHL using the so-called Petri Net (PN) simulation networks [12]. Their work, inspired by a previous theoretical model of cellular oxygen-related pathways [13] [14], was a preliminary investigation of the core oxygen sensing system and its connection with VHL onset. Heiner and coworkers proposed three different functional modules responsible for hypoxia network control and for HIF-1α degradation [12]. In other words, they theorized that hereditary forms of cancer, such as different manifestations of VHL, are the result of different and concomitantly compromised metabolic pathways.

### Von Hippel-Lindau Disease

Von Hippel-Lindau protein (pVHL) is the product of the von Hippel-Lindau gene, located in the short arm of 3rd chromosome, and constantly transcribed in both fetal and adult tissues [15]. Mutations of pVHL are related to a pathological outcome termed VHL syndrome, an inherited form of cancer [16]. VHL syndrome is characterized by cysts and tumors growing in specific parts of the organism [16]–[17]. It is considered a severe autosomal dominant genetic condition with inheritance of one person in over 35,000 [18]. The tumor injuries, which can be either benign or malign, are usually located in the retina, adrenal glands, epididymis, central nervous system, kidneys and pancreas [19]. As a genetic disorder, VHL syndrome follows Knudson’s two hit principle. A copy of the gene is mutated in the germ line, but the other gene copy still produces a functional protein. Complete protein inactivation appears during life due to somatic inactivation of the remaining functional copy [20]. On the contrary, mutations occurring during early fetal formation result in unsuccessful development [21]. The pVHL gene has 11,213 base pairs including three exons [18] and the final transcript is a protein commonly present in two isoforms: pVHL30 and pVHL19, of 213 and 160 residues respectively. Neither isoform contains a known enzymatic domain, but rather appears to serve as a multipurpose adapter protein engaging in multiple protein-protein interactions [22]. pVHL structure is organized in an α- and β-domain and its stability was demonstrated to be ensured by direct interaction with other proteins such as Elongins B and C [23]. Both Elongin B and C are also required for the best characterized function of pVHL, the ubiquitination dependent degradation of Hypoxia Inducible Factor (HIF) via the proteasome [24]. However, pVHL is considered a multipurpose protein due to its high number of known interactors. At the time of writing, the IntAct database [25] presents more than 200 different interaction partners, with some of them competing for the same Elongin binding site. Indeed, pVHL was found in different cellular compartments and seems to be involved in many different cellular processes such as apoptosis, cell proliferation, survival and motility [26]. Considering the huge number of interactors and multiple cellular localizations, many different functions have been described or hypothesized, such as regulation of cytoplasmic microtubules during mitosis [27] and endothelial extracellular matrix deposition [28]. On the other hand, considering the huge number of players involved in VHL syndrome and the lack of reliable kinetic data, a PN based approach may be a preferable option for an entire VHL pathway simulation.

### Petri Net for Interaction Pathways

Since their invention, by Carl Adam Petri in the early sixties, PNs were mostly used to describe technical systems, but later the utility in describing biological and biochemical functions has also been demonstrated [29]. PNs were successfully used in many studies to describe biological networks [30], such as the regulation and etiopathology in human Duchenne Muscular Dystrophy [31] and the hypoxia response network [12]. PNs are qualitative mathematical models that can graphically represent many object types, not only metabolites but also different protein states and are useful to simulate networks where not only metabolites are involved. Indeed, PNs can be a powerful tool to study all concurrent interactions in a specific pathway, even if the proteins or kinetics are not well-known. Due to the large number of different pVHL functions involved in VHL disease progression, we decided to extend the PN based analysis of [12] increasing the number of considered protein-protein interactions. We generated a novel manually curated PN model of the entire VHL regulation system collecting data from the literature and including the signaling pathways and glucidic metabolism. In order to build a realistic network, literature from both biochemical experiments and *in silico* predictions were used as source. It was decided to build a PN with only confirmed pVHL interactions whose function was also known. The resulting PN was validated using an analysis of specific properties as suggested by previous studies using the same method [29]. After validating the PN structure, *in silico* knock outs of specific proteins were done in order to observe the different network behaviors and the resulting biological effect.

## Methods

The network was designed in the Snoopy PN framework (version 2, revision 1.13) [32], respecting the mathematical PN formalism as described in [12]–[33]. PN were demonstrated to be useful in describing discrete and concurrent processes in a simple graphical representation [30] and have been used to describe biomedical processes due to their capacity of representing sequential steps in a process. PN modeling methods are actively used to describe, simulate, analyze, and predict the behavior of biological systems. The Snoopy PN framework provides an extensible multi-platform framework to design, animate, and simulate Petri nets [32]. We chose Snoopy to facilitate future extensions of the VHL pathway presented here. Among different available PN types a standard PN was chosen to limit the number of variables. Both Charlie and PInA analyzers were used for PN analysis and validation (34). Further, *in silico* knock out experiments were used to test the biological reliability of the model. Structural model validation was made by analysis of the T-invariants to demonstrate whether the system was covered by T-invariants and to confirm the biological meaning of each invariant. The use of T- and P-invariants is given by their own properties: they are a set (of transitions or places, respectively) that allow the reproduction of the same state after *n* transformations. A P-invariant represents a set of places where the number of tokens is constant and independent on the firing rate. A T-invariant instead represents a set of transitions that cyclically comes back to show the same initial set. Biologically a P invariant can represent the process of regulating a protein, whereas T invariants can represent cyclical biochemical transformations such as metabolic reactions. To this end, the computed invariants were grouped in Maximal Common Transition Sets (MCTS) and Clusters, the former based on occurrence of specific sets of transition inside the various T-invariants, and the latter based on similarities between T-invariants. Different numbers of clusters will be defined depending on the resulting square matrix. Where MCTS create disjunctive nets, Clusters merge together similar T-invariants. Behavioral validation was made by selectively deleting tokens inside the model, imitating possible biological disruptions such as disease-causing mutations. The resulting network behavior was compared to what is reported in the literature. Total runtime for invariants computation were less than ten seconds on a mainstream Linux x86 workstation. Literature sources used to build the model are reported in Table S1. The Snoopy framework for PN construction, Charlie and PInA tools for analysis are available at the website (URL: http://www-dssz.informatik.tu-cottbus.de/DSSZ/Software). Finally, the model was used to simulate the network behavior through visual inspection of both token movement and accumulation in specific parts of the network. For a visual explanation of token movement in a PN refer to Video S1.

### Model Availability

The resulting VHL disease PN model is available in File S1.

## Results

### Notations and Assumptions

**Figure 1:**
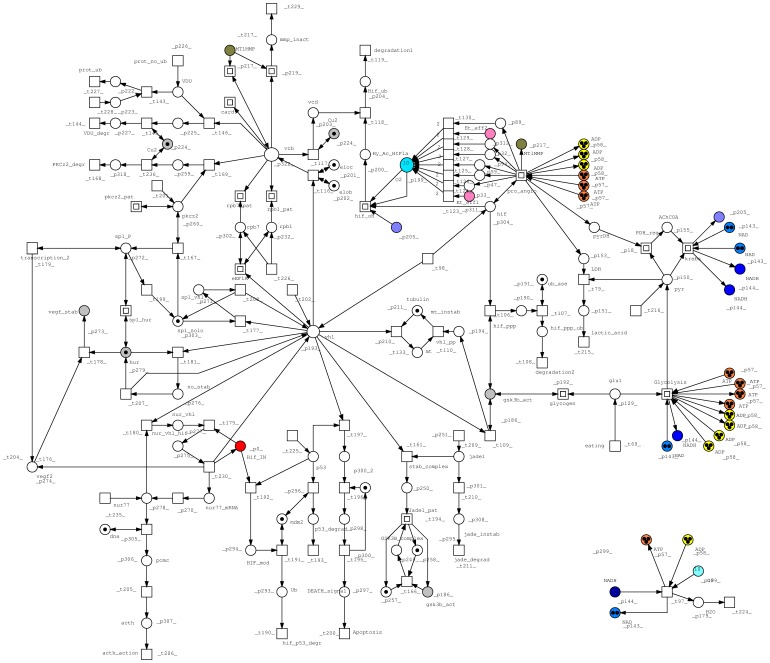
Top level model. The colors of some tokens were arbitrarily chosen to give a clearer identification of the central nodes (ATP, Vcb and oxygen) or for nodes involved in more reactions such as GSK3β. The group of nodes in the bottom left is not disconnected from the central body of the network thanks to the presence of logic nodes for ATP synthesis (t_97).

**Figure 2:**
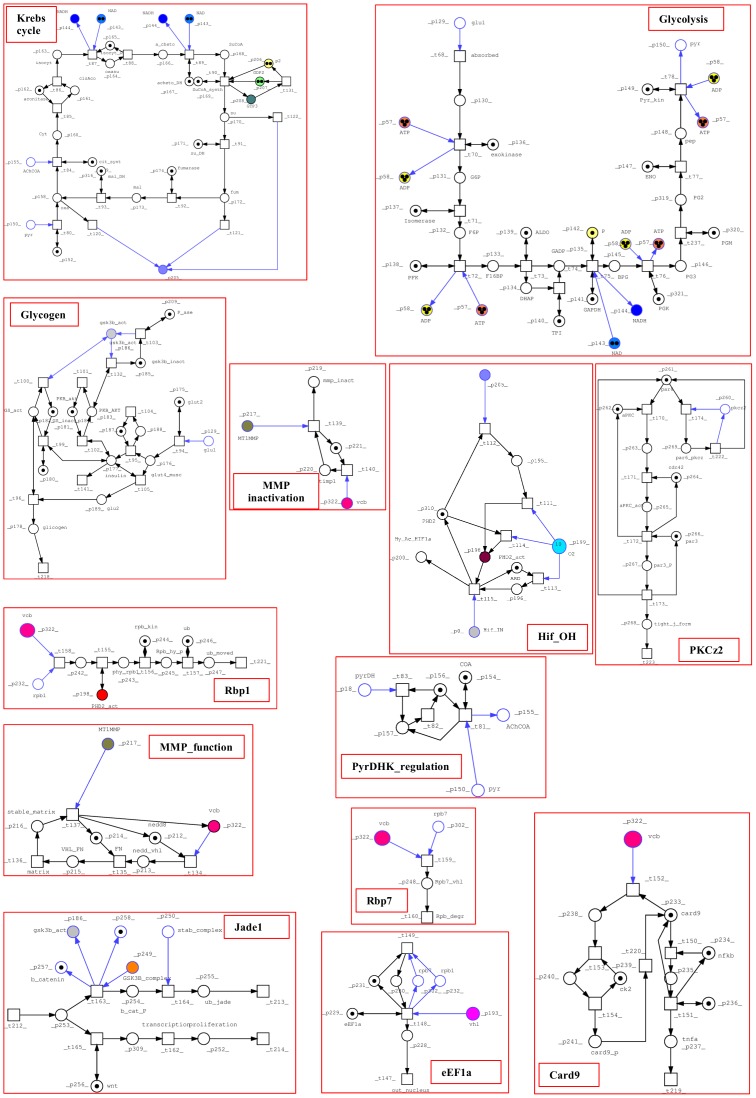
Lower hierarchical PN levels. Pathways from the top level are grouped in macro-nodes (functional subordinated layer), in particular glucidic metabolism and various VHL functions.

**Figure 3:**
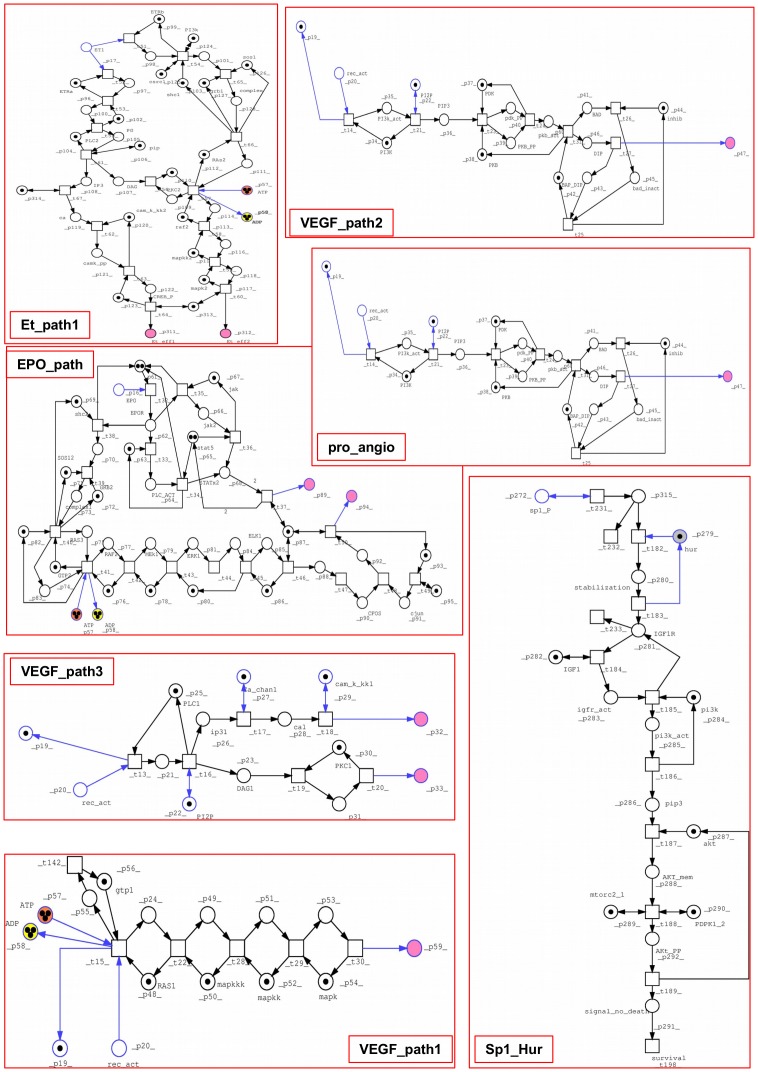
Lower hierarchical PN levels, in particular HIF-1α regulation and HIF-1α-dependent pro-angiogenic signaling. VEGF and EPO pathways are at a lower hierarchical level than the pro_angio macro-node.

The PN built here focuses on pVHL interactions that were already proven by biochemical experiments and reported in the literature. We chose to model a realistic VHL disease pathways based on confirmed literature data, including all known VHL functions, VHL related signal pathway and glucidic metabolism. All bibliographic sources used to design the model are presented in Table S1. The final PN is composed of 323 places and 238 transitions, connected by 801 arcs. Tables S1 and S2 show all places and transitions and the related biological correspondence. Places are mainly proteins and enzymes, while some represent DNA or small molecular substrates such as glucose and cofactors (e.g. ATP). Notation for both pre- and post-places and their biological meaning are explained in Table S1. In a few cases, places are used to represent a whole group of changes generated by DNA transcription, (e.g. p_32 and p_33 or Et_eff1 and Et_eff2). Transitions instead symbolize complex formation between two proteins or post-translational modifications. Output transitions stand for degradation or movement to other parts of the cell or organism to complete their functions (e.g. degrad_1 and degrad_2) whereas input transitions show the generation of a substrate or protein. In order to simplify the design of such a large network, we decided to use macro nodes to group reactions representing complex molecular pathways such as signaling pathways or secondary signal cascades. The whole process is merged into a single node with a given name to allow visual inspection only in case of need. From the top level all transitions can still be found in a hierarchical lower layout level. Logic nodes were used for places participating in many reactions throughout the network such as ATP and ADP (7 logical copies each) or NAD and NADH (4 logical copies each). A total nesting depth of two was chosen to model macro nodes. Special arcs were not used while we chose to model the permanent presence of some objects using double arcs (e.g. for elob, eloc and places standing for enzymatic activity). In case of proteins which are actively degraded, it was preferred to create an input transition simulating constant production (or synthesis) and an output for consumption. This is the case for pkcz2, Jade1, pVHL and HIF-1α. As can be seen from Figures 1 to 3, which represent the entire model, two major nodes can be immediately identified: pVHL and vcb, the complex made by pVHL and the two elongins. Another relevant part is the glucidic metabolism, modeled due to its hypoxia induced regulation. It is represented in detail in Figure 2.

### HIF-1α Transcription Activity

The HIF-1α transcription factor stimulates proliferation of endothelial cells to create new blood vessels during localized or broad hypoxia. In human, it is present as three different paralogs: HIF-1α, HIF-2α and HIF-3α. The sequence is quite conserved between the former two, whereas the latter is slightly shorter and seems to have completely different functions compared to the other two [33]–[34]. Both HIF-1α and -2α stimulate DNA transcription but the exact products of this activity are still poorly understood. In our model, only HIF-1α *in vivo* activity was considered. It cannot be excluded that other biological effects depend on the second paralog. Indeed, both have a pro-angiogenetic function and are degraded by pVHL via proline-directed hydroxylation. HIF is a heterodimer of HIF-1α and HIF-1β, the latter being also termed Aryl hydrocarbon Receptor Nuclear Translocator (ARNT). We started from the transcription activity of HIF due to its regulation is the most studied pVHL function. Our model, as expected from literature data, shows that HIF-1α enters the nucleus when not degraded by pVHL. It subsequently binds HIF-1β to form the HIF heterocomplex which interacts with DNA. Our model correctly simulates the increased affinity of HIF towards DNA. Transcription is enhanced by some co-factors binding both subunits of HIF and other proteins such as p300, Creb and cjun. This takes place in a specific DNA promoter sequence termed Hypoxia Response Element (HRE). Furthermore, during transcription some pro-angiogenic factors are produced: Vascular Endothelial Growth Factor (VEGF), Endothelin (ET) and Erythropoietin (EPO). All described pathways are in agreement with previous observations reported in [35].

### Metabolic Processes

HIF-1α transcription activity includes some proteins which are dependent on oxygen but involved in other pathways (e.g. oxidative metabolism) or completely independent (e.g. metallo-proteinase MT1MMP). Further, HIF-1α stimulates production of proteins involved in the glucidic pathway. The final product of the metabolism is adenosine triphosphate (ATP), a molecular form of energy, composed by adenosine, an adenine ring connected to a ribose sugar, and three phosphate moieties. When a phosphate moiety is hydrolyzed it releases energy, used by cells for enzymatic reactions. The glucidic metabolism is composed of glycolysis, Krebs cycle, glycogen formation and respiratory chain with ATP synthesis. Glucose is absorbed in cells by enzymatic glucose transporters (GLUT), which carry the molecule to the location inside the cell where the metabolism takes place [36]. There are many isoforms of these transporters: GLUT1 is present in all cells and in particular in erythrocytic membranes, neurons and glia [37]. GLUT2, located in both liver and pancreatic beta cells, is characterized by low affinity for glucose, hence it requires a higher glucose concentration to be activated [38]. Right after eating, glucose concentration increases, thereby quickly activating them. GLUT2 stimulates production of insulin, a hormone regulating the plasmatic glucose concentration. Glucose plasmatic concentration can also increase due to an opposite pathway, originating from liver glycogen being decomposed into glucose and reaching systemic circulation. GLUT3 is mostly present in neurons, whereas GLUT4 is the insulin activated transporter located in myocytes, adipocytes and cardiomyocytes [36]–[39]. In our model, we chose to exclude GLUT3 due to its specific role in neuronal cells. Glycolysis occurs in the cytoplasm and during this process each glucose molecule is phosphorylated, consuming two molecules of ATP, then divided into two smaller molecules. Further modifications of these two molecules result in new ATP production. The molecule obtained at the end of glycolysis is pyruvate, which can be again modified through three different pathways. It can be decarboxylated and linked to Co-enzyme A to form acetyl-Co-enzyme A. It can then be carboxylated to obtain oxalacetate, or transformed through lactate dehydrogenase into lactic acid. Pyruvate can also be generated by other metabolic pathways, like protein or fatty acid disruption and amino-acid modifications. Acetyl-CoA and oxalacetate are the molecules used in the following glucidic metabolism process, the Krebs cycle, taking place in the mitochondrial matrix. The Krebs cycle starts with acetyl-CoA and oxalacetate merging to create citric acid, which continues undergoing modifications until oxalacetate is formed again. During the process some co-enzymes are modified. Decarboxylation of pyruvate to form acetyl-CoA already transforms a NAD+ (Nicotinamide Adenine Dinucleotide) in NADH (reduced form), afterwards obtaining one more of ATP, GTP, FADH_2_ (Flavin Adenine Dinucleotide) and three more NADH per pyruvate molecule entering the Krebs cycle. The redox co-enzymes are considered electron transporters. During metabolic reactions they reduce themselves and get electrons (and protons) to oxidize the substrate of the enzymatic reaction. Electrons taken during the glucose metabolism are then used in the respiratory chain taking place in the internal mitochondrial membrane. The respiratory chain consists in transporting electrons through enzymes called cytochromes and others co-enzymes, characterized by the capability to receive and donate electrons. NADH (FADH_2_) is oxidized again by cytochromes going back to the form of NAD (or FAD). Electrons gained through oxidation are used to reduce half a molecule of oxygen into water, releasing more energy. The FADH_2_ and NADH redox chain establishes a chemical potential causing the push of protons outside the internal membrane towards the inter-membrane space, which stays between the mitochondrial inner and outer membrane. This also causes a higher concentration of protons outside the inner membrane. The resulting gradient causes the tendency of protons to enter the cell. The final step is ATP-synthetase, formed by a channel that allows protons to enter, pushed by the gradient, allowing the enzyme to change conformation and make its reaction. This kinetic energy is converted into ATP. In our model, the glycolytic and Krebs cycles were described in detail, represented at the hierarchical second level by the coarse transition Glycolysis. The respiratory chain was instead merged into a single node (t_97). We chose to represent creation and consumption of ATP in order to show the effects of lower and higher oxygen concentration on the network. On the other hand, oxygen consumption for ATP synthesis during the respiratory chain creates a flow of oxygen in the model. Oxygen is not the only connection between glucidic metabolism and hypoxia. Indeed, HIF-1α transcription activity enhances the transcription of many GLUT isoforms (such as 1, 3 and 9) and the pyruvate dehydrogenase kinase, which determines the pyruvate dehydrogenase (PyrDH) inactivation and consequent Acetyl-CoA formation from pyruvate. Finally, Lactate dehydrogenase is also produced, to ensure an alternative compound, creating energy needed for cell survival [36]–[40].

### pVHL-dependent Processes

Some interactors can bind pVHL in regions interacting with Elongin C. These are HuR, Nur77, p53 and Jade1. Nur77 has a complex function and its role in pVHL tumor suppressor activity is still not entirely clear. Nur77 can bind pVHL, inhibiting Elongin binding while allowing HIF-1α binding. Its transcription is stimulated by HIF-1α itself, and pVHL-HIF-1α-Nur77 complex formation stabilizes the transcription activity of HIF-1α by inhibiting the pVHL-dependent degradation [41]. Another Nur77 function is the stimulation of proopiomelanocortin (POMC) transcription, which is a precursor for adrenocorticotropic hormone (ACTH) formation. This hormone has an important stress response function, stimulating cortisol production and other neurotransmitters from the adrenal glands, to enhance the organism reaction to danger and stress *stimuli* e.g. increase of gluconeogenesis and muscle mass. An excess of this hormone can cause desensitization of its receptors for feedback down-regulation and thus muscular weakness, tiredness, hyperglycemia and osteoporosis [42]. p53 can bind to pVHL avoiding the degradation of this tumor suppressor. Instead, it stimulates the apoptotic signal cascade via the p300 co-activator, which stimulates production of proteins enhancing the cell programmed death. If p53 cannot bind pVHL, two more mechanisms are described in the model. One is its modification and degradation by Mdm2 and the other is the pVHL-independent degradation of HIF-1α. Interaction with Mdm2 is needed in both cases [43]–[44]. Jade1 is a short-lived protein whose main function is to stimulate the phosphorylation-dependent degradation of β-catenin. This is a subunit of the cadherin protein complex acting as an intracellular signal transducer in the Wnt signaling pathway. It seems that β-catenin is able to stop cell division via a contact-dependent inhibition signal, whereas in Wnt signaling it is also involved in proliferative transcription. When Wnt is not present, β-catenin can be phosphorylated by Glycogen Synthetase Kinase, type 3β (GSK3β) in complex with APC (Adenomatous Polyposis Coli) and Axin. β-catenin can interact with Jade1 and be only successfully degraded after this interaction [45]. Related functions are represented in the macro node Jade1_pat. GSK3β seems to be a protein involved in many different pathways. GSK3β is involved in Glycogen Synthetase deactivation and can even phosphorylate pVHL and HIF-1α. In the case of HIF-1α, it generates a pVHL-independent degradation pathway, where phosphorylation allows ubiquitination, whereas in the case of pVHL, it inhibits pVHL stabilization of microtubules [46].

### Structural Model Analysis

Based also on previous observations of Heiner et al., [47], in 2008 Grunwald et al., demonstrated that PN can be used to describe large and complex metabolic pathways [31]. They postulated the following set of minimal rules that a PN should satisfy to be considered biologically reliable: (1) the network should be entirely connected, (2) the network should be covered by T-invariants, and (3) each T-invariant and P-invariant should have a biological meaning. The model described here was tested with respect to what previously done by Grunwald and co-workers [31] and resulted to be covered by T-invariants, connected, homogeneous and each place has a pre-transition and a post-transition. Transitions without pre- or post-places were used to simulate the system interface to the surroundings. The network is alive, in other words, it continues to work forever, with all transitions contributing to the net behavior forever, and no dead transitions. The MCTS and Cluster analysis were used due to the large number of T- and P-invariants included in the model. Both methods are used in PN theory to reduce the complexity connected with such a large network and to reduce the errors connected with manual investigation. From the 238 transitions present at the beginning in the model, 393 T-invariants were computed without considering 10 trivial invariants. The latter consist in a pair of transitions that usually represent a forward and backward reaction, such as the active and inactive state of a protein. Trivial invariants could be erased to reduce the dimension of the network without disturbing the overall system when the interest is focused on the steady state behavior [12]. T-invariants were grouped into 44 Clusters using the Tanimoto coefficient with similarity threshold of 65%, as described in [31]. Only 11 of these 44 comprised more than one T-invariant. The three biggest Clusters are C9, composed of 144 T-invariants, C8 of 72 and C11 of 64 T-invariants. Separation into clusters allows easier analysis of networks pathways represented by each T-invariant, since they are grouped by similarity, specifically the common transitions by which they are composed. T-invariants named in the text are shown in Table S3, while T-invariants grouped in C8, C9, C10, C11 are explained in Table S4 and described as follows.

### Cluster C8

Cluster C8 groups all transitions included in HIF-1α pathways, including transcription, signaling cascades, degradation via pVHL, p53 and GSK3β, and eventually the Krebs cycle. For the EPO signaling pathway, two transitions (t_35 and t_36) are not included which cause Jak activation and consequent Stat5 activation to stimulate DNA transcription. Matrix stability regulation is also part of the cluster due to the destabilization induced by HIF-1α transcription of metallo-proteinase (MMP), transitions from t_134 to t_140. The largest T-invariant in C8 is Inv_280 (93 transitions) while the smallest is Inv_377 (81 transitions). The differences between T-invariants show the possibility of alternative pathways inside the model. For example, the VEGF dependent signal cascade can proceed in three different ways: t_13, t_14 and t_15, which lead to the pathways being merged in the coarse nodes Vegf_path3, Vegf_path2 and Vegf_path1, respectively. The occurrence rate in C8 is 24 transitions for each path. The Endothelin, VEGF and Erythropoietin pathways are not in conflict and occurring together. Disaggregation of the matrix via MMPs is present in 18 T-invariants, whereas inhibition of these proteins, i.e. matrix stabilization, is present in the remaining 54 transitions. Regarding the Krebs cycle, 47 T-invariants have t_91, of which only 24 reach t_92 and t_93, representing the last three steps of the cycle: succinate to fumarate, fumarate to malate, and malate to oxalacetate. All the malate being produced is used to regenerate oxalacetate. Degradation of HIF-1α occurs in any T-invariant of the cluster. The pVHL-dependent degradation of HIF-1α is always present (transitions t_116 to t_119). In 19 T-invariants degradation takes place via p53 (t_191 to t_193) or, alternatively, via phosphorylation by GSK3β in another 17 T- invariants. Two of the three pathways can be present in the same T-invariant, as in Inv_227, where degradation via pVHL and degradation via p53 are both present. This was considered as the HIF-1α dependence on the lack of degradation by these proteins. All three degradation pathways never appear in the same T-invariant. The p53 and GSK3β paths are never present together but each of them is accompanied by pVHL-dependent proteasomal degradation. Inv_377 lacks the EPO signaling pathway but is the only one in this cluster to have t_34, t_33 and t_37. These invariants have all input and output transitions. For example, t_202 the second input for pVHL, is present in only 18 invariants. Other inputs are t_98, always present, leading to formation of HIF-1α and pVHL, t_192, producing p53 and t_216, representing other pyruvate generating metabolic pathways. The latter is also present in each invariant allowing formation of the pyruvate needed for Krebs cycle progression.

### Cluster C9

Cluster C9 is the largest cluster in our model and includes 144 T-invariants. It is characterized by complete EPO pathway abrogation which goes through formation of the Shc-Grb-Sos complex and the consequent mapk-dependent phosphorylation cascade. Transition t_127, representing EPO effects on oxygen production, is absent. In its place, t_35 and t_36 are considered, which are present in 72 T-invariants. In cluster C9, the largest T-invariants are Inv_278 and Inv_279 (74 transitions) while the shortest ones are Inv_101, Inv_105, Inv_144 and Inv_148 with 65 transitions each.

### Cluster C10

Cluster C10, composed of 52 T-invariants, is characterized by the presence of glycolysis between many transitions grouped in the cluster. This is also the cluster containing the most populated T-invariant of all computed 393 non-trivial T-invariants. This is Inv_245, including 101 transitions and covering almost half of the whole model. Cluster C10 also includes Inv_125, the shortest invariant of this model, composed by 85 transitions due to lack of the Krebs cycle. Another difference with the other three major clusters is that here both EPO paths are present, specifically, the Jak pathway belongs to 4 T-invariants and Shc-Grb-Sos is observed throughout the cluster. Vegf_path1 seems to be more common in this cluster, being present in 36 T-invariants, whereas the other two are present 12 times each. This time they are present even in the same invariant, as for Inv_60, Inv_129, Inv_172 and Inv_215, with both t_13 and t_15, and Inv_142, Inv_185 and Inv_228 with t_14 and t_15 and all subsequent signaling appearing at the same time. Despite glycolysis being present in all cluster invariants, the Krebs cycle appears only in 11 cases. p53-dependent degradation of HIF-1α occurs in 11 cases while the phosphorylation-dependent one appears in 13. An input transition has been added with respect to the other major clusters so far analyzed (i.e. t_69_eating) without which glycolysis could never take place.

### Cluster C11

**Figure 4:**
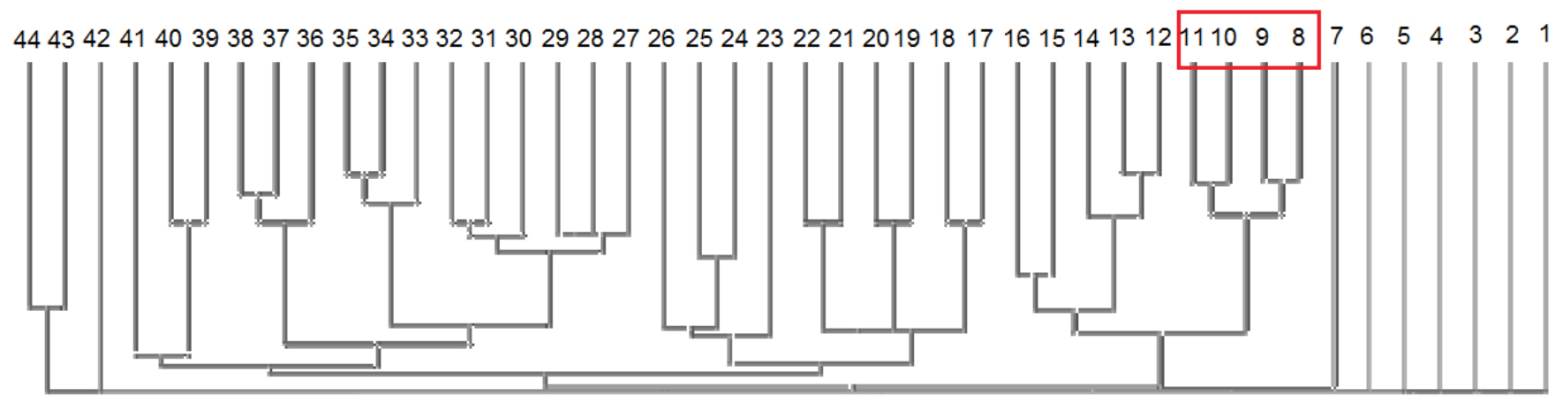
PinA Distance Matrix clustering, using Tanimoto coefficient and 65% threshold of. The numbers indicates clusters. In C8, C9, C10, C11 are highlighted a red square.

Cluster C11 is composed of 64 T-invariants. Only part of the EPO pathway is described here, with the major difference that the Krebs cycle is completely abrogated while Prolyl Hydroxylase type 2 (PHD2) regulation by oxalacetate is included. HIF-1α interaction with Nur77 and transcription of VEGF by Sp1 are also present. t_80 (transformation of pyruvate in oxalacetate) is not present in the first 42 cluster T-invariants. Nur77 interaction with HIF-1α is present only in 8 T-invariants, specifically Inv_87 to Inv_94. Sp1 transcription activity is appearing in twice the amount, including the same 8 invariants just mentioned. VEGF transcription via Sp1 activity is aPKCζ2 phosphorylation dependent, which does however not appear in the cluster. When VEGF is synthesized, it is subsequently stabilized by Hur, followed by t_178 and Hur is recreated to allow other functions. Indeed, it is one of the few places without input transition but with a token that goes forward and backward again. Compared to the other clusters, C11 also shows one less transition in the coarse PHD regulation node, specifically t_81, which shows the transformation of pyruvate by pyrDH into acetyl-Coenzyme A, needed for the Krebs cycle. The four clusters C-8 to C12 are very similar to each other, as can be seen from the distance tree in Figure 4. They all contain the HIF-1α transcription activity and signaling pathways caused by EPO, VEGF and the HIF-1α degradation options. They include the effects of other transcription activity products, like metallo-proteinase and pyruvate dehydrogenase kinase, which regulate the activation state of PyrDH. All include part of the glucidic metabolism but not Glycogen formation itself. Other five clusters from C12 to C16 have a smaller number of T-invariants and fewer transitions present in each invariant. They do not include transcription activity but are only formed by the VEGF and glycolytic pathways. The information contents of these clusters turned out to be uninformative and their analysis was not included. The same applies to clusters composed by 1–3 T-invariants. Finally, some transitions are not present in the clusters and not listed in the T-invariants because trivial invariants were excluded from cluster analysis. These transitions are shown in Table 1 with their respective biological meaning.

**Table 1:** List of Trivial T-invariants excluded from calculation with their associated biological meaning.

Trivial T-Invariants	ID transitions	Biological Meaning
TInv_1	t_99, t_100	Glycongen Synthase regulation
TInv_2	t_101, t_102	Pkb regulation
TInv_3	t_103, t_132	GSK3β active-inactive state
TInv_4	t_174, t_222	Par6 inactivation via aPKCζ2
TInv_5	t_177, t_208	VHL binding to Sp1
TInv_6	t_167, t_199	Sp1 phosphorylation and dephosphorylation
TInv_7	t_0, t_2	Hif transport in and out of nucleus
TInv_8	t_0, t_234	Hif inhibition via FIH
TInv_9	t_181, t_207	Hur inhibition via VHL
TInv_10	t_231, t_232	IGFR mRNA production and destruction

### MCTS Analysis

**Figure 5:**
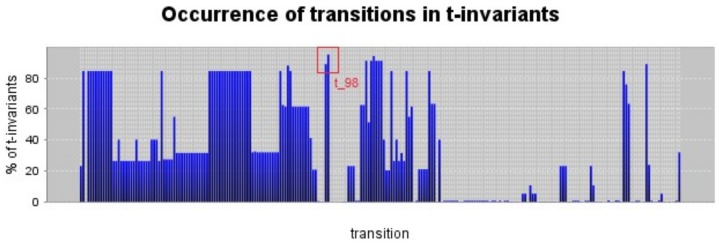
Transitions occurrence T-invariants. Transitions are ordered by name and t_98 is highlighted in red.

Another way to group invariants is by the amount of single transitions present in them. Maximal common transition set (MCTS) analysis provides a PN decomposition into non-overlapping subnets, sharing parts of the same T-invariants [29]. In a biochemical network, MCTS could be interpreted as enzyme subsets operating together under steady state conditions, computed based on the support of a T-invariant. MCTS computation does not consider stoichiometric relations, describing exclusively sets of reactions present in a maximal number of T-invariants resulting shared by different signaling pathways [48]. A total of 40 non-trivial MCTS were identified, with results and related biological means shown in Table 2. Some transitions do not belong to a non-trivial MCTS, because their occurrence has no similarity with other transitions and they create separate MCTS (specifically: t_69, t_82, t_91, t_94, t_98, t_114, t_116, t_120, t_121, t_122, t_179, t_202, t_209, t_212, t_216, t_225 and t_229). MCTS define transitions that always take place together, but are not necessarily connected, thus representing disjunct building blocks constituting the network. Considering both analyses, a table was automatically built in PInA [29] showing a correlation between clusters and MCTS. Transitions (t) or MCTS (M) are compared to evaluate how many T-invariants clusters cover the selected M or t (if the transition is not already part of the MCTS, as listed above). The more covered a transition or set is, the more central it could be considered for the network behavior. Recently, a network coarsening method based on abstract dependent transition sets (ADT) was presented [49]. It is formulated without the requirement of pre-computation of the T-invariants and is a tool commonly used for the decomposition of large biochemical networks into smaller subnets. Due to the manually designed nature of our model, we preferred to maintain a logic hierarchy based on metabolic pathways in order to maintain the network centered on pVHL and its interaction. The MCTS calculation results shows that the most covered set by cluster T-invariants is M20 with 358 T-invariants covering all transitions in the set, indicating that this MCTS corresponds to more T-invariants than the others. All transition sets are an important link to the others, as tokens pass through these transitions more often. A transition not present in any set but most covered by T-invariants is t_98, which is also the most frequently occurring transition, see Figure 5. The 10 most occurring transitions are listed in the Table 3.

**Table 2:** List of MCTS and transitions from PInA.

MCTS	ID Transitions
MCTS 1 (M1)	t_0, t_190, t_191, t_192;
MCTS 2 (M2)	t_1, t_3, t_4, t_5, t_6, t_7, t_8, t_9, t_10, t_11, t_12, t_32, t_51, t_52, t_53, t_54, t_55, t_56, t_57, t_58, t_59, t_60, t_61, t_62, t_63, t_64, t_65, t_66, t_67, t_79, t_83, t_123, t_129, t_138, t_215;
MCTS 3 (M3)	t_2, t_99, t_100, t_101, t_102, t_103, t_132, t_167, t_174, t_181, t_199,_177, t_207, t_208, t_222, t_232, t_234;
MCTS 4 (M4)	t_13, t_16, t_17, t_18, t_19, t_20, t_124, t_128;
MCTS 5 (M5)	t_14, t_21, t_23, t_24, t_25, t_26, t_27, t_31, t_126;
MCTS 6 (M6)	t_15, t_22, t_28, t_29, t_30, t_125, t_142;
MCTS 7 (M7)	t_33, t_34;
MCTS 8 (M8)	t_35, t_36;
MCTS 9 (M9)	t_37, t_130;
MCTS 10 (M10)	t_38, t_39, t_40, t_41, t_42, t_43, t_44, t_45, t_46, t_47, t_48, t_49, t_50, t_127;
MCTS 11 (M11)	t_68, t_70, t_71, t_72, t_73, t_74, t_75, t_76, t_77, t_78, t_237;
MCTS 12 (M12)	t_80, t_111, t_112;
MCTS 13 (M13)	t_81, t_84, t_85, t_86, t_87, t_88, t_89, t_90, t_131;
MCTS 14 (M14)	t_92, t_93;
MCTS 15 (M15)	t_95, t_104, t_141;
MCTS 16 (M16)	t_96, t_105, t_218;
MCTS 17 (M17)	t_97, t_224;
MCTS 18 (M18)	t_106, t_107, t_108;
MCTS 19 (M19)	t_109, t_110, t_133;
MCTS 20 (M20)	t_113, t_115, t_117, t_118, t_119;
MCTS 21 (M21)	t_134, t_135, t_136, t_137;
MCTS 22 (M22)	t_139, t_140, t_217;
MCTS 23 (M23)	t_143, t_227, t_228;
MCTS 24 (M24)	t_144, t_145, t_146;
MCTS 25 (M25)	t_147, t_148, t_149;
MCTS 26 (M26)	t_150, t_151, t_219;
MCTS 27 (M27)	t_152, t_153, t_154, t_220;
MCTS 28 (M28)	t_155, t_156, t_157, t_158, t_159, t_160, t_221, t_226;
MCTS 29 (M29)	t_161, t_163, t_164, t_166, t_213;
MCTS 30 (M30)	t_162, t_165, t_214;
MCTS 31 (M31)	t_168, t_169, t_201, t_236;
MCTS 32 (M32)	t_170, t_171, t_172, t_173, t_223;
MCTS 33 (M33)	t_175, t_176, t_180, t_230;
MCTS 34 (M34)	t_178, t_203;
MCTS 35 (M35)	t_182, t_183, t_231, t_233;
MCTS 36 (M36)	t_184, t_185, t_186, t_187, t_188, t_189, t_198;
MCTS 37 (M37)	t_193, t_194;
MCTS 38 (M38)	t_195, t_196, t_197, t_200;
MCTS 39 (M39)	t_204, t_205, t_206, t_235;
MCTS 40 (M40)	t_210, t_211;

**Table 3:** Ranking of the 10 most occurring transitions with biological meaning and percentage of occurrence.

Rank	Transitions	Biological meaning	Occurrence %
1	t_98	Input transition for Hif and VHL	95.165
2	t_116	Interaction of VHL with Elongin B and C	94.148
3	t_113	Activation by oxygen of ARD	94.094
4	t_115	Acetylation and hydroxilation of Hif	94.094
5	t_117	Interaction of complex Vcb with Cu2	94.094
6	t_118	Interaction of complex Vcb with modified Hif	94.094
7	t_119	Degradation VHL dependent of Hif	94.094
8	t_97	ATP formation	89.059
9	t_224	Water Output transition	89.059
10	t_82	Pyruvate Dehydrogenase inactivation	88.041

### P-invariant Analysis

Although the network is not covered by P-invariants, it has 130 P-invariants. 47 of these are trivial P-invariants, comprising a single place, connected with double arcs to imitate an activator arc function. Another object represented with double arcs is the enzymatic activity catalyzing a reaction and immediately going back to the steady state. P-invariants show places or sets of places where token numbers always remain equal and do not move outside the subnetwork induced by the P-invariant in the initial marking. In other words, they do not grow nor diminish. The remaining P-invariants are mostly located in signal transduction pathways, such as situations in which a protein is sequestered from its function and then goes back after a second reactivation mechanism. This scenario is present in p_41, p_42 and p_45 located in invariant P_58. It is important to notice that ATP and ADP, as well as NAD and NADH, are modeled as P-invariants. P_90, P_91 and t_97 are able to transform ATP and ADP. More in general, all energy consuming transitions are considered to be backward transitions of invariants. Invariants not related to signal transduction are places located in the Hur system, where Hur is removed from its function by pVHL. This is a good approximation for sequential modifications that momentarily activate proteins. Afterwards, Hur can go back and stabilize VEGF to increase its transcription activity.

### In Silico Knock Out Experiments

The previously described clustering and MCTS analysis for T-invariants allowed us to identify the most common transitions and to understand which transitions can be depleted in our knock out experiments in order to get the most important biological effect. The knock out experiments were performed erasing selected transitions or tokens and observing which transitions or MCTS become inactivated. Considering our results and the literature, we decided to knock out the following pathway elements: (i) pVHL, (ii) HIF1α alone and with Sp1, (iii) t_98, (iv) PHD2, (v) MCTS1, (vi) t_97 and (vii) GSK3β. In the following, we describe the effect of each knock out scenario on our model.

#### (i) pVHL knock out

Degradation of HIF-1α is not completely depleted due to presence of both p53- and GSK3β-dependent alternative degradation pathways. All other processes usually inhibited by pVHL take place in an uncontrolled way, including creation of VEGF via Sp1 transcription activity and increased matrix regulation due to lack of fibronectin crosslinking. Hur resulted constantly activated and nur77 can stimulate synthesis of Proopiomelanocortin, precursor for the Adrenocorticotropic hormone. Card9 increases release of tumor necrosis factor, and NF-kB when not inhibited by pVHL. Instead, Jade1 is unable to survive long enough to inhibit β−catenin, generating a proliferation signal with Wnt. Lactic acid is also not produced due to LDH enzyme production being HIF-1α transcription activity dependent.

#### (ii) HIF-1α knock out

VEGF is still created thanks to Sp1, thus oxygen is still generated even if in lower proportion. If HIF-1α and Sp1 are both knocked out at the same time, oxygen is quickly consumed and the metabolism is soon unable to proceed. Lactic acid is not produced due to LDH enzyme production being HIF-1α transcription activity dependent. Glycolysis and glycogen are produced normally and the metabolism is not inhibited by PyrDH negative regulation and lactic acid formation. Since pVHL is present, other tumor suppressor activities are enabled, except for proteasomal degradation of HIF-1α due to the substrate being non-existent.

#### (iii) HIF-1α and pVHL double knock out

This generates a situation where the metabolism is normal but oxygen regeneration is less productive, with only Sp1 acting for transcription. Due to absence of pVHL, all proliferation-stimulating processes are active, causing an unbalanced consumption of resources. Our model shows that this condition is compatible with cell growth and multiplication, but new blood vessel generation is consistently slower and glucidic metabolism appears principally based on the glycolysis reaction. Similar activity reduction applies to both tight junction and cellular external matrix (ECM) pathway regulation. It cannot be excluded that some observed effects could be mitigated by both HIF-2α and HIF-3α activity *in vivo.*

#### (iv) PHD2 knock out

The protein is involved in pVHL mediated and oxygen dependent degradation of HIF-1α. Further, PHD2 is involved in hydroxylation of the RNA polymerase II subunit Rpb1 to allow its translocation to less chromatin-concentrated areas of the nucleus. When it is knocked out, HIF-1α degradation can continue via alternative pathways as seen in the pVHL knock out experiment and there is more RNA polymerase II activity, even if rpb7 can still be inactivated by pVHL.

#### (v) MCTS1 knock out

MCTS1 groups some reactions involved in the HIF-1α p53-dependent degradation pathway (Table 2). To perform this knock out, we erased the necessary token in mdm2, making the precondition insufficient to enable the MCTS transitions. p53 is not degraded and can continue its proapoptotic signal. On the other hand, a HIF-1α degradation mechanism is also knocked out resulting in an increased HIF-1α transcription activity.

#### (vi) t_97 knock out

This is the ATPase transition, allowing the model to imitate oxygen consumption for ATP synthesis. If this transition is inactive, oxygen accumulates infinitely and ATP is not regenerated after few simulation steps. At the beginning, ATP is formed during the first step of glycolysis but afterwards it is consumed again. At some point, these reactions do not have any ATP available to allow the system to re-balance the consumed ATP. After few simulation steps, oxygen reaches a high level due to slower consumption in the PHD2 regulation process. Biologically, this means that the metabolism stops and the cell is not able to create energy to survive. There is no accumulation other than glucose in the model. A few oxygen creation processes are blocked as well due to absence of ATP, e.g. t_15, t_41 and t_57.

#### (vii) GSK3β knock out

This enzyme is involved in negative glycogen synthetase (GS) regulation and is inactivated when phosphorylated. When GSK3β is knocked out, glycogen is continuously produced due to the enzyme remaining in an active state. In a real organism there are alternative forms of GSK3β which can inactivate GS, hence the effect will be less sharp. GSK3β is also involved in the degradation of HIF-1α, causing its phosphorylation and following ubiquitination. It is also involved in the degradation of β-catenin, where it is responsible for primary phosphorylation. If knocked out, even if Jade1 can be stabilized by pVHL, the effect will be similar to a knock out of Jade1, where β-catenin is free to continue proliferation stimulating transcription activity.

## Discussion

We started from a core model of hypoxia response [12] and extended the original network with functional data derived from the literature in order to represent a complete description of the pVHL interaction pathway according to current knowledge. VHL syndrome is characterized by the formation of tumors and cysts affecting different organism districts and tissues. Indeed, pVHL is a tumor suppressor whose functions are connected to inhibition of proliferation and survival, growth and stability of extracellular matrix and microtubules, as well as cell polarity and migration. The IntAct database reports more than 200 suspected pVHL interactors and for most of them interaction and function details remain largely unknown. We chose to model the pVHL interactions in a credible cellular context with many protein activities occurring at the same time. The main idea was to create a novel manually curated PN description of the entire VHL disease pathway, including glucidic metabolism and signaling pathways. The model was designed as a standard PN and is composed of 238 transitions and 323 places, connected by 801 edges. A biologically realistic PN model needs to be covered by T-invariants, meaning each transition in the model has to be included in a T-invariant, and each invariant needs to have a biological meaning [31]–[47]. We used the T-invariant analysis to validate the reliability of the model. We computed a total of 393 T-invariants, plus 10 trivial invariants, which were excluded from analysis. These were grouped into 44 Clusters and, through use of T-invariants, transitions were grouped into 40 MCTS. The model obtained is connected, covered by T-invariants with each invariant holding a biological meaning. MCTS analysis was used to identify the most frequent crucial transitions occurring in the model. This specific subset was further used to plan *in silico* knock out experiments and for the model validation and analysis of expected biological behavior. The model was then used to perform *in silico* knock out experiments inactivating specific transitions during qualitative network analysis. Our results showed that the model is able to represent important transitions reflecting real biological outcomes, i.e. transitions involving species such as oxygen or ATP are correctly inactivated under certain circumstances as expected from the bibliographic data. Biological energy-related reactions (e.g. ATP production from ADP) were modeled as P-invariants. Although the network is intentionally not covered by P-invariants, P-invariant analysis was used to verify all modeled energy consuming transitions. Both the ATP and NADH balances appeared constant during the simulation, with irrelevant P-invariants located in the Hur system. This approximation was used to verify the Hur-dependent regulation of VEGF, with results in accordance with [50]. The specific pVHL knock out suggests that this protein alone is not sufficient for complete HIF-1α inactivation. Indeed, other concurrent HIF-1α degradation pathways promote a sort of cell cycle regulation backup. On the contrary, simple deletion of pVHL turned out to be sufficient to increase all its other inhibitory functions, showing similar effects to pathological VHL symptoms. Indeed, ECM destabilization increases cell migration to other areas, promoting metastasis outbreak in case of tumor cells. Further, pVHL-dependent inhibition of tight junction formation by aPKCζII participates in an easier cellular detachment. The interactions of Nur77 could be considered a good example for pathological effects. It is a stimulator of Proopiomelanocortin production, a precursor for the Adrenocorticotropic hormone. If excessively released, it promotes an overproduction of adrenergic neurotransmitters by adrenal glands. Coming at clinical condition known as Cushing syndrome. On the very long term, Nur77 deregulation is known to cause tumors of the pituitary and adrenal glands [51], [52]. This happens in pheochromocytoma, which is one of the main VHL disease manifestations. We speculate that continuous VEGF transcription, even in situations where HIF-1α (but not Sp1) is knocked out, could be the explanation for clinical studies where VEGF-targeting drugs have turned out to be effective in kidney cancer treatment as reported in [53]. Although we used only confirmed data from the literature, Nur77 may be involved in other regulation systems which were not considered in our model. The transitions for pVHL fibronectin stabilization show a behaviour which is coherent with biochemical experiments, illustrating a complete abrogation of ECM stabilization and an increased matrix metallo-proteinase action. Although the results are encouraging, the presented model will need further improvements since standard PNs do neither allow a complete transition control nor enzymatic activity modulation. Nevertheless, thanks to its manual curation our model can be used to plan new *in vitro* and *in vivo* experiments. The results are convincing enough to suggest our model as a comprehensive pathway model to simulate the main pVHL functions.

## Supporting Information

Table S1 **List of model transitions.** The sequential number, name, biological functions and bibliographic source are listed.
(XLS)

Table S2 **List of all model places.** The progressive ID number, name and biological meaning are shown.
(PDF)

Table S3 **List of T-invariants named in the text and their composition.**
(PDF)

Table S4 **Composition in terms of invariants of the clusters C8, C9, C10, C11.**
(PDF)

File S1 **VHL disease network model in spped format.**
(SPPED)

Video S1 **Snoopy PN framework running demo.**
(MP4)
