# Supplementary material for: Design and Analysis of a Petri Net Model of the Von Hippel-Lindau (VHL) Tumor Suppressor Interaction Network
Source: PLoS One. 2014 Jun 2;9(6):e96986. doi: 10.1371/journal.pone.0096986 (PMC4041725; doi:10.1371/journal.pone.0096986)
Supplement: Table S2 — List of all model places. The progressive ID number, name and biological meaning are shown. (PDF) [file pone.0096986.s002.pdf]

Place Number	Name	Biological Meaning
p_0	Hif_IN	HIF1α after entering nucleus
p_1	ARNT	Hif1β/ARNT
p_2	HIF_dim	Dimeric form of HIF
p_3	p300	Coactivator p300
p_4	creb	Coactivator CREB
p_5	cjun1	Coactivator cjun
p_6	Creb_300_HIF	HIF-p300-CREB complex
p_7	HIF_act	completely activated form of HIF
p_8	hre	Hypoxia Response Element
p_9	_place_9	DNA transcripted
p_10	_place_10	ET_mRNA
p_11	_place_11	VEGF_mRNA
p_12	_place_12	EPO_mRNA
p_13	_place_13	Lactate DH mRNA
p_14	_place_14	pyrDH Kinase_mRNA
p_15	VEGF	VEGF peptide
p_16	EPO	EPO peptide
p_17	ET1	ET peptide
p_18	pyrDH	PyrDH protein
p_19	_place_19	VEGF receptor
p_20	rec_act	activated VEGF receptor
p_21	_place_21	PLC1 activated
p_22	PI2P	Phosphatydil inositol biphosphate
p_23	DAG1	Diacylglycerol
p_24	_place_24	RAS1activated
p_25	PLC1	Phospholipase C
p_26	ip31	Ip3, 1 to avoid non-unique names
p_27	Ca_chan1	calcium Channel
p_28	ca1	Calcium Ion
p_29	cam_k_kk1	Calmodulin Kinase system
p_30	PKC1	Protein Kinase C
p_31	_place_31	PKC1 activated
p_32	_place_32	VEGF signal pathway 3 effects
p_33	_place_33	VEGF signal pathway 3 effects
p_34	PI3K	Phosphoinositide 3 kinase
p_35	PI3k_act	Phosphoinositide 3 kinase-active state
p_36	PIP3	Phosphoinositol 3 phosphate
p_37	PDK	Phosphoinositide dependent kinase
p_38	PKB	Protein Kinasi B also known as Akt
p_39	PKB_PP	PKB phosphorilated
p_40	pdk_PP	PDK phosphorilated
p_41	BAD	B Apoptosis Domain
p_42	BAP_DIP	complex BAD and DIP
p_43	_place_43	DIP after action

Place Number	Name	Biological Meaning
p_44	inhib	BAD inhibitor protein
p_45	bad_inact	BAD inactive state
p_46	DIP	DIP - PBAP Chromatin Remodeling protein
p_47	_place_47	Vegf signal pathway 2 effects
p_48	RAS1	RAS GTPase
p_49	_place_49	Mapkkk inactive
p_50	mapkkk	mapkkk active
p_51	_place_51	mapkk inactive
p_52	mapkk	mapkk active
p_53	_place_53	mapk inactive
p_54	mapk	active mapk
p_55	gdp1	Guanosine Diphosphate
p_56	gtp1	Guanosine Triphosphate
p_57	ATP	Adenosine Triphosphate
p_58	ADP	Adenosine Diphosphate
p_59	_place_59	Vegf signal pathway 1 effects
p_60	pkb_act	Protein Kinase B – active state
p_61	_place_61	Inactive EPO receptor
p_62	EPOR	active EPO Receptor
p_63	_place_63	inactive PLC
p_64	PLC_ACT	Phospholipase C – active state
p_65	stat5	transcriptor factor
p_66	jak2	transcriptor factor
p_67	jak	transcriptor factor
p_68	STATx2	dimer form of stat5
p_69	shc2	Src homology containing protein
p_70	_place_70	active shc
p_71	SOS12	Son of Sevenless protein
p_72	GRB2	Growth factor receptor-bound protein 2
p_73	complex1	Shc-sos-grb complex
p_74	GTP2	Guanosine Triphosphate
p_75	RAS3	RAS GTP-ase
p_76	_place_76	inactive RAF2, serine/threonine kinase
p_77	RAF2	RAF2 serine/threonine kinase
p_78	_place_78	inactive mek1
p_79	MEk1	Mitogen-activated protein kinase kinase
p_80	_place_80	inactive ERK1
p_81	ERK1	Extracellular signal-regulated kinases
p_82	_place_82	inactive RAS3
p_83	_place_83	GDP - Guanosine diphosphate
p_84	_place_84	ERK in nucleus
p_85	ELK1	ETS domain-containing protein
p_86	_place_86	ELK1 inactive
p_87	_place_87	DNA
p_88	_place_88	CFOS mRNA
p_89	_place_89	EPO effects1
p_90	CFOS	Transcription factor
p_91	cjun	Transcription factor

Place Number	Name	Biological Meaning
p_92	_place_92	Cfos, cjun complex
p_93	_place_93	inactive cjun
p_94	_place_94	EPO effects2
p_95	_place_95	cjun activating protein
p_96	ETRa	Endothelin Receptor a
p_97	_place_97	ETRa active
p_98	_place_98	ETRa2 active
p_99	ETRb	Endothelin Receptor b
p_100	_place_100	Protein G - active
p_101	_place_101	shc active state
p_102	PG	Protein G
p_103	shc1	Src homology containing protein
p_104	PLC2	Phospholipase C
p_105	_place_105	PLC2 active
p_106	pip	Phosphatinositol phosphate
p_107	DAG	diacylglycerol
p_108	IP3	Inositol triphosphate
p_109	PKC2	Protein kinase C
p_110	_place_110	PKC2 active state
p_111	_place_111	Ras2 active state
p_112	RAs2	Ras
p_113	raf2	RAF2 serine/threonine kinase
p_114	_place_114	raf2 active state
p_115	mapkk2	Mitosis activated protein kinase kinase
p_116	_place_116	Mapkk2 active state
p_117	mapk2	Mitosis activated protein kinase
p_118	_place_118	mapk2 active state
p_119	ca	Calcium ion
p_120	cam_k_kk2	Calcium-calmodulin-kinase complex
p_121	camk_pp	Calmodulin kinase complex phosphorilated state
p_122	CREB_P	CREB phosphorilated
p_123	_place_123	CREB
p_124	PI3k	Phosphoinositide 3 kinase
p_125	csrc1	SRC
p_126	sos1	Son of sevenless
p_127	grb1	GRB
p_128	complex	Sos-shc-grb complex
p_129	glu1	glucose
p_130	_place_130	glucose inside cells
p_131	G6P	Glucose 6 Phosphate
p_132	F6P	Fructose 6 phosphate
p_133	F16BP	Fructose 1,6- diphosphate
p_134	DHAP	duhydroxyacetone phosphate
p_135	GADP	D-glyceraldehyde 3 phosphate
p_136	exokinase	Hexokinase enzime
p_137	Isomerase	Glucose-6-phosphate isomerase
p_138	PFK	Phopshofructokinase
p_139	ALDO	Aldolase

Place Number	Name	Biological Meaning
p_140	TPI	triosephosphato isomerase
p_141	GAPDH	glyceraldehyde phosphate dehydrogenase
p_142	P	Pyrophosphate
p_143	NAD	
p_144	NADH	
p_145	BPG	Biphosphoglycerate
p_146	PG3	3phosphoglycerate
p_147	ENO	
p_148	pep	Phophoenolpyruvate
p_149	Pyr_kin	Pyruvate kinase
p_150	pyr	Pyruvate
p_151	lactic_acid	
p_152	_place_152	Pyruvate carboxylase
p_153	LDH	Lactate dehydrogenase
p_154	COA	Coenzyme A
p_155	AChCOA	Acetyl coenzyme A
p_156	_place_156	active pyrdh
p_157	_place_157	inactive pyr dh
p_158	oxa	Oxalacetate
p_159	cit_synt	citrate s
p_160	Cyt	Cytrate
p_161	cisAco	Cis-aconitate
p_162	aconitase	
p_163	isocyt	Isocytrate
p_164	oxasu	oxalosuccinate
p_165	isocyt_DH	isocitrate dehydrogenase
p_166	a_cheto	A-ketoglutarate
p_167	acheto_DH	A-ketoglutarate dehydrogenase
p_168	SuCoA	Succinil-coA
p_169	SuCoA_synth	Succinil-coA synthetase
p_170	su	Succinate
p_171	su_DH	Succinate Dehydrogenase
p_172	fum	Fumarate
p_173	mal	Malate
p_174	fumarase	
p_175	glut2	GLUT2
p_176	_place_176	glucose inside pancreas cells
p_177	insulin	
p_178	glicogen	
p_179	H2O	water
p_180	_place_180	phosphatase inactivating GS
p_181	GS_inact	inactive Glycogen synthetase
p_182	GS_act	active Glycogen synthetase
p_183	PKB_AKT	Protein Kinase B aka AKT
p_184	PKB_akt	inactive PKB
p_185	gsk3b_inact	Glycogen Synthetase Kinase 3b inactive
p_186	gsk3b_act	active glycogen synthetase kinase 3b

Place Number	Name	Biological Meaning
p_187	_place_187	pancreatic insulin synthesis complex
p_188	_place_188	active insulin synthesis system
p_189	glu2	Glucose inside muscles and fat cells
p_190	hif_ppp	Phosphorylated HIF
p_191	ub_ase	Ubiquitinating system
p_192	hif_ppp_ub	Phosphorylated and ubiquitinated hif
p_193	vhl	Von Hippel Lindau
p_194	vhl_pp	Phosphorylated VHL
p_195	_place_195	PHD2 inactivated by Krebs cycle analogues
p_196	ARD	Aryl Ribonuclease domain
p_197	_place_197	active ARD
p_198	PHD2_act	active PHD2
p_199	O2	oxygen
p_200	Hy_Ac_HIF1a	Modified HIF
p_201	eloc	Elongin C
p_202	elob	Elongin B
p_203	vcd	complex of VHL elongins and Cu2-ring box
p_204	Hif_ub	HIF after ubiquitination
p_205	_place_205	inhibiting analogues of oxoglutarate for PHD2
p_206	p2	phosphate
p_207	GDP2	Guanosine diphosphate
p_208	GTP3	Guanosine triphosphate
p_209	P_ase	Phosphatase
p_210	mt	Microtubules
p_211	tubulin	Tubulin
p_212	nedd8	Nedd8
p_213	nedd_vhl	Neddylated VHL
p_214	FN	Fibronectine
p_215	VHL_FN	complex of Vhl with Fibronectine
p_216	stable_matrix	crossed linked matrix
p_217	MT1MMP	Metallo proteinase 1
p_218	_place_218	metallo proteinase mRNA
p_219	mmp_inact	inactivated MMP
p_220	timp1	Tissue inhibiting Metallo proteinase1
p_221	_place_221	inactive Tissue inhibiting metallo proteinase1
p_222	prot_no_ub	deubiquitinated generic protein
p_223	prot_ub	ubiquitinated protein
p_224	Cu2	cullin2
p_225	_place_225	complex VDU and vcb
p_226	VDU	VHL interactiong deubiquitinase
p_227	_place_227	Ubiquitinated VDU
p_228	_place_228	Vhl moved out of nucleus
p_229	eEF1a	Endonuclear export farctor1a
p_230	_place_230	Functional Rna polimerase
p_231	_place_231	rna polimarase other subunits
p_232	rpb1	Rna polimerasi binding protein *subunit1
p_233	card9	Caspase associated recruiting domain

Place Number	Name	Biological Meaning
p_234	nfkb	NF-kB
p_235	_place_235	activated nfkb+card9
p_236	_place_236	transcription activity
p_237	tnfa	tumor necrosis factor a
p_238	_place_238	complex VCB card9
p_239	ck2	Kinase activity on Card9
p_240	_place_240	ck2 inactive
p_241	card9_p	card9 phosphorylated
p_242	_place_242	Rbp-vhl
p_243	phy_rpb1	Prolin-hydroxylated RPB1
p_244	rpb_kin	Rpb kinase
p_245	Rpb_hy_p	Rpb- hydroxilated and phosphorilated form
p_246	ub	ubiquitin
p_247	ub_moved	rpb1-hydroxphosphorilated-ubiquitinated and moved out of nucleus
p_248	Rpb7_vhl	Rpb subunit 7 in complex with vhl
p_249	GSK3B_complex	Glicogen sintase kinase 3 b complex with
p_250	stab_complex	Stable jade complex
p_251	jade1	jade
p_252	proliferation	effect
p_253	b_catenin	Beta catenin
p_254	b_cat_P	Beta catenin phosphorilated form
p_255	ub_jade	Jade ubiquitinated
p_256	wnt	Signaling Pathways: Wnt / β-Catenin Signaling
p_257	_place_257	APC
p_258	_place_258	Axin
p_259	_place_259	VHL in complex with PKCzII
p_260	pkcz2	Atipical Protein kinase C zeta II
p_261	par6	Subunit of tight junction par 6
p_262	aPKC	Atipical protein kinase C unknown type
p_263	_place_263	Par6 in complex with aPKC
p_264	cdc42	CDC42 is a protein involved in regulation of the cell cycle
p_265	aPKC_act	Atypical PKC unknown type- active state
p_266	par3	Subunit of tight junction par 3
p_267	par3_P	Par 3 phosphorilated form
p_268	tight_j_form	Formation of tight junction
p_269	par6_pkcz	Complex of pkcz with par 6
p_270	nur77_mRNA	MRNA of protein nur77 nuclear receptor
p_271	sp1_vhl	Sp1 transcriptor factor in complex with vhl
p_272	sp1_P	Sp1 phosphorilated form
p_273	vegf_stab	Stable VEGF
p_274	vegf2	Additional vegf to increase action of HIF-induced
p_275	nur_vhl_hif	Complex nur 77 with HIF and VHL
p_276	no_stab	instability
p_277	nur_vhl	Complex of vhl and nur77
p_278	nur77	Nuclear receptor family 4 type 1a
p_279	hur	hur RNA-binding protein
p_280	stabilization	Stabilization of IGFR mRNA

Place Number	Name	Biological Meaning
p_281	IGF1R	Insulin like growth factor receptor
p_282	IGF1	Insulin like growth factor 1
p_283	igfr_act	Insulin like growth factor receptor – active state
p_284	pi3k	Phosphoinositide 3 kinase
p_285	pi3k_act	Phosphoinositide 3 kinase – active state
p_286	pip3	Phosphatinositol phosphate
p_287	akt	PKB - Akt, also known as Protein Kinase B (PKB)
p_288	AKT_mem	PKB activated with PIP3
p_289	mtorc2_1	Mtorc protein
p_290	PDPK1_2	Prolin Directed protein Kinase
p_291	signal_no_death	Signal for survival
p_292	AKt_PP	PKB phosphorylated
p_293	Ub	ubiquitin
p_294	HIF_mod	Modified Hif by p53
p_295	_place_295	P53 and Mdm2, towards degradation
p_296	mdm2	MDM2binding domain
p_297	DEATH_signal	Signal for apoptosis
p_298	_place_298	p300 and p53 complex
p_299	p300_2	Coactivator of transcription p300
p_300	_place_300	p53 and vhl complex
p_301	p53	Tumorsuppressor p53
p_302	rpb7	Rna polimerase subunit 7
p_303	sp1_solo	Sp1 alone
p_304	hif	Hipoxia inducible factor 1a
p_305	dna	Desoxy ribonucleic acid
p_306	pomc	Proopiomelanocortin
p_307	acth	Adenocorticotropic Hormone
p_308	jade_instab	Jade – unstable form
p_309	_place_309	bcatenin activated by wnt
p_310	PHD2	Prolin Hydroxilating Domain containing Protein 2
p_311	Et_eff1	Endotelin effects 1 vasocostriction
p_312	Et_eff2	Endotelin effects 2 vasocostriction high blood pressure
p_313	_place_313	DNA in et1
p_314	_place_314	ca channel in et
p_315	_place_315	IGFR mRNA
p_316	mal_DH	Malate dehydrogenase
p_317	FIH	Factor inhibiting hif
p_318	_place_318	ubiquitinated pkcz2
p_319	PG2	2-phosphoglycerate
p_320	PGM	Phosphoglycerate mutase
p_321	PGK	Phophoglycerate kinase
p_322	vcb	VHL in complex with elongins B and C
