# Supplementary material for: Design and Analysis of a Petri Net Model of the Von Hippel-Lindau (VHL) Tumor Suppressor Interaction Network
Source: PLoS One. 2014 Jun 2;9(6):e96986. doi: 10.1371/journal.pone.0096986 (PMC4041725; doi:10.1371/journal.pone.0096986)
Supplement: Table S3 — List of T-invariants named in the text and their composition. (PDF) [file pone.0096986.s003.pdf]

# Inv	Transitions
Inv_60	1, 3, 4, 5, 6, 7, 8, 9, 10, 11, 12, 13, 15, 16, 17, 18, 19, 20, 22, 28, 29, 30, 32, 38, 39, 40, 41, 42, 43, 44, 45, 46, 47, 48, 49, 50, 51, 52, 53, 54, 55, 56, 57, 58, 59, 60, 61, 62, 63, 64, 65, 66, 67, 68.absorbed, 69.eating, 70, 71, 72, 73, 74, 75, 76, 77, 78, 79, 82, 83, 97, 98, 113, 114, 115, 116, 117, 118, 119.degradation1, 123, 124, 125, 127, 128, 129, 134, 135, 136.matrix, 137, 138, 142, 215, 224, 237
Inv_87	1, 3, 4, 5, 6, 7, 8, 9, 10, 11, 12, 15, 22, 28, 29, 30, 32, 33, 34, 37, 51, 52, 53, 54, 55, 56, 57, 58, 59, 60, 61, 62, 63, 64, 65, 66, 67, 68.absorbed, 69.eating, 70, 71, 72, 73, 74, 75, 76, 77, 78, 79, 82, 83, 97, 98, 113, 114, 115, 116, 117, 118, 119.degradation1, 123, 125, 129, 130, 134, 135, 136.matrix, 137, 138, 142, 175, 176, 178, 180, 203, 215, 224, 230, 237,
Inv_88	1, 3, 4, 5, 6, 7, 8, 9, 10, 11, 12, 15, 22, 28, 29, 30, 32, 35, 36, 37, 51, 52, 53, 54, 55, 56, 57, 58, 59, 60, 61, 62, 63, 64, 65, 66, 67, 68.absorbed, 69.eating, 70, 71, 72, 73, 74, 75, 76, 77, 78, 79, 82, 83, 97, 98, 113, 114, , 115, 116, 117, 118, 119.degradation1, 123, 125, 129, 130, 134, 135, 136.matrix, 137, 138, 142, 175, 176, 178, 180, 203, 215, 224, 230, 237
Inv_89	1, 3, 4, 5, 6, 7, 8, 9, 10, 11, 12, 15, 22, 28, 29, 30, 32, 33, 34, 37, 51, 52, 53, 54, 55, 56, 57, 58, 59, 60, 61, 62, 63, 64, 65, 66, 67, 68.absorbed, 69.eating, 70, 71, 72, 73, 74, 75, 76, 77, 78, 79, 82, 83, 97, 98, 113, 114, 115, 116, 117, 118, 119.degradation1, 123, 125, 129, 130, 138, 139, 140, 142, 175, 176, 178, 180, 202, 203, 215, 217, 224, 230, 237
Inv_90	1, 3, 4, 5, 6, 7, 8, 9, 10, 11, 12, 15, 22, 28, 29, 30, 32, 35, 36, 37, 51, 52, 53, 54, 55, 56, 57, 58, 59, 60, 61, 62, 63, 64, 65, 66, 67, 68.absorbed, 69.eating, 70, 71, 72, 73, 74, 75, 76, 77, 78, 79, 82, 83, 97, 98, 113, 114, 115, 116, 117, 118, 119.degradation1, 123, 125, 129, 130, 138, 139, 140, 142, 175, 176, 178, 180, 202, 203, 215, 217, 224, 230, 237
Inv_91	1, 3, 4, 5, 6, 7, 8, 9, 10, 11, 12, 15, 22, 28, 29, 30, 32, 33, 34, 37, 51, 52, 53, 54, 55, 56, 57, 58, 59, 60, 61, 62, 63, 64, 65, 66, 67, 68.absorbed, 69.eating, 70, 71, 72, 73, 74, 75, 76, 77, 78, 79, 82, 83, 97, 98, 106, 107, 108.degradation2, 113, 114, 115, 116, 117, 118, 119.degradation1, 123, 125,

# Inv	Transitions
	129, 130, 138, 139, 140, 142, 175, 176, 178, 180, 203, 215, 217, 224, 230, 237
Inv_92	1, 3, 4, 5, 6, 7, 8, 9, 10, 11, 12, 15, 22, 28, 29, 30, 32, 35, 36, 37, 51, 52, 53, 54, 55, 56, 57, 58, 59, 60, 61, 62, 63, 64, 65, 66, 67, 68.absorbed, 69.eating, 70, 71, 72, 73, 74, 75, 76, 77, 78, 79, 82, 83, 97, 98, 106, 107, 108.degradation2, 113, 114, 115, 116, 117, 118, 119.degradation1, 123, 125, 129, 130, 138, 139, 140, 142, 175, 176, 178, 180, 203, 215, 217, 224, 230, 237
Inv_93	0, 1, 3, 4, 5, 6, 7, 8, 9, 10, 11, 12, 15, 22, 28, 29, 30, 32, 33, 34, 37, 51, 52, 53, 54, 55, 56, 57, 58, 59, 60, 61, 62, 63, 64, 65, 66, 67, 68.absorbed, 69.eating, 70, 71, 72, 73, 74, 75, 76, 77, 78, 79, 82, 83, 97, 98, 113, 114, 115, 116, 117, 118, 119.degradation1, 123, 125, 129, 130, 138, 139, 140, 142, 175, 176, 178, 180, 190.hif_p53_degr, 191, 192, 203, 215, 217, 224, 225, 230, 237,
Inv_94	0, 1, 3, 4, 5, 6, 7, 8, 9, 10, 11, 12, 15, 22, 28, 29, 30, 32, 35, 36, 37, 51, 52, 53, 54, 55, 56, 57, 58, 59, 60, 61, 62, 63, 64, 65, 66, 67, 68.absorbed, 69.eating, 70, 71, 72, 73, 74, 75, 76, 77, 78, 79, 82, 83, 97, 98, 113, 114, 115, 116, 117, 118, 119.degradation1, 123, 125, 129, 130, 138, 139, 140, 142, 175, 176, 178, 180, 190.hif_p53_degr, 191, 192, 203, 215, 217, 224, 225, 230, 237
Inv_101	1, 3, 4, 5, 6, 7, 8, 9, 10, 11, 12, 15, 22, 28, 29, 30, 32, 33, 34, 37, 51, 52, 53, 54, 55, 56, 57, 58, 59, 60, 61, 62, 63, 64, 65, 66, 67, 79, 81, 82, 83, 84, 85, 86, 87, 88, 89, 90, 91, 92, 93, 97, 98, 113, 114, 115, 116, 117, 118, 119.degradation1, 123, 125, 129, 130, 131, 134, 135, 136.matrix, 137, 138, 142, 215, 216, 224
Inv_105	1, 3, 4, 5, 6, 7, 8, 9, 10, 11, 12, 15, 22, 28, 29, 30, 32, 35, 36, 37, 51, 52, 53, 54, 55, 56, 57, 58, 59, 60, 61, 62, 63, 64, 65, 66, 67, 79, 81, 82, 83, 84, 85, 86, 87, 88, 89, 90, 91, 92, 93, 97, 98, 113, 114, 115, 116, 117, 118, 119.degradation1, 123, 125, 129, 130, 131, 134, 135, 136.matrix, 137, 138, 142, 215, 216, 224
Inv_125	1, 3, 4, 5, 6, 7, 8, 9, 10, 11, 12, 13, 16, 17, 18, 19, 20, 32, 38, 39, 40, 41, 42, 43, 44, 45, 46, 47, 48, 49, 50, 51, 52, 53, 54, 55, 56, 57, 58, 59, 60, 61, 62, 63, 64, 65, 66, 67, 68.absorbed, 69.eating, , 70, 71, 72, 73, 74, 75, 76, 77, 78, 79, 82, 83, 97, 98, 113, 114, 115, 116, 117, 118, 119.degradation1, 123, 124, 127, 128, 129, 138, 139, 140, 202, 215, 216, 217, 224, 237
Inv_129	1, 3, 4, 5, 6, 7, 8, 9, 10, 11, 12, 13, 15, 16, 17, 18, 19, 20, 22, 28, 29, 30, 32, 38, 39, 40, 41, 42, 43, 44, 45, 46, 47, 48, 49, 50, 51, 52, 53, 54, 55, 56, 57, 58, 59, 60, 61, 62, 63, 64, 65, 66, 67, 68.absorbed, 69.eating, 70, 71, 72, 73, 74, 75, 76, 77, 78, 79, 82, 83, 97, 98, 113, 114, 115, 116, 117, 118, 119.degradation1, 123, 124, 125, 127, 128, 129, 138, 139, 140, 142, 202, 215, 217, 224, 237
Inv_142	1, 3, 4, 5, 6, 7, 8, 9, 10, 11, 12, 14, 15, 21, 22, 23, 24, 25, 26, 27, 28, 29, 30, 31, 32, 38, 39, 40, 41, 42, 43, 44, 45, 46, 47, 48, 49, 50, 51, 52, 53, 54, 55, 56, 57, 58, 59, 60, 61, 62, 63, 64, 65, 66, 67, 68.absorbed, 69.eating, 70, 71, 72, 73, 74, 75, 76, 77, 78, 79, 82, 83, 97, 98, 113, 114, 115, 116, 117, 118, 119.degradation1, 123, 125, 126, 127, 129, 138, 139, 140, 142, 202, 215, 217, 224, 237
Inv_144	1, 3, 4, 5, 6, 7, 8, 9, 10, 11, 12, 15, 22, 28, 29, 30, 32, 33, 34, 37, 51, 52, 53, 54, 55, 56, 57, 58, 59, 60, 61, 62, 63, 64, 65, 66, 67, 79, 81, 82, 83, 84, 85, 86, 87, 88, 89, 90, 91, 92, 93, 97, 98, 113, 114, 115, 116, 117, 118, 119.degradation1, 123, 125, 129, 130, 131, 138, 139, 140, 142, 202, 215, 216, 217, 224
Inv_148	1, 3, 4, 5, 6, 7, 8, 9, 10, 11, 12, 15, 22, 28, 29, 30, 32, 35, 36, 37, 51, 52, 53, 54, 55, 56, 57, 58, 59, 60, 61, 62, 63, 64, 65, 66, 67, 79, 81, 82, 83, 84, 85, 86, 87, 88, 89, 90, 91, 92, 93, 97, 98, 113, 114, 115, 116, 117, 118, 119.degradation1, 123, 125, 129, 130, 131, 138, 139, 140, 142, 202, 215, 216, 217, 224
Inv_172	1, 3, 4, 5, 6, 7, 8, 9, 10, 11, 12, 13, 15, 16, 17, 18, 19, 20, 22, 28, 29, 30, 32, 38, 39, 40, 41, 42, 43, 44, 45, 46, 47, 48, 49, 50, 51, 52, 53, 54, 55, 56, 57, 58, 59, 60, 61, 62, 63, 64, 65, 66, 67, 68.absorbed, 69.eating, 70, 71, 72, 73, 74, 75, 76, 77, 78, 79, 82, 83, 97, 98, 106, 107, 108.degradation2, 113, 114, 115, 116, 117, 118, 119.degradation1, 123, 124, 125, 127, 128, 129, 138, 139, 140, 142, 215, 217, 224, 237
Inv_185	1, 3, 4, 5, 6, 7, 8, 9, 10, 11, 12, 14, 15, 21, 22, 23, 24, 25, 26, 27, 28, 29, 30, 31, 32, 38, 39, 40, 41, 42, 43, 44, 45, 46, 47, 48, 49, 50, 51, 52, 53, 54, 55, 56, 57, 58, 59, 60, 61, 62, 63, 64, 65, 66, 67, 68.absorbed, 69.eating, 70, 71, 72, 73, 74, 75, 76, 77, 78, 79, 82, 83, 97, 98, 106, 107, 108.degradation2, 113, 114, 115, 116, 117, 118, 119.degradation1, 123, 125, 126, 127, 129, 138,

# Inv	Transitions
	139, 140, 142, 215, 217, 224, 237
Inv_215	0, 1, 3, 4, 5, 6, 7, 8, 9, 10, 11, 12, 13, 15, 16, 17, 18, 19, 20, 22, 28, 29, 30, 32, 38, 39, 40, 41, 42, 43, 44, 45, 46, 47, 48, 49, 50, 51, 52, 53, 54, 55, 56, 57, 58, 59, 60, 61, 62, 63, 64, 65, 66, 67, 68.absorbed, 69.eating, 70, 71, 72, 73, 74, 75, 76, 77, 78, 79, 82, 83, 97, 98, 113, 114, 115, 116, 117, 118, 119.degradation1, 123, 124, 125, 127, 128, 129, 138, 139, 140, 142, 190.hif_p53_degr, 191, 192, 215, 217, 224, 225, 237
Inv_227	0, 1, 3, 4, 5, 6, 7, 8, 9, 10, 11, 12, 14, 21, 23, 24, 25, 26, 27, 31, 32, 38, 39, 40, 41, 42, 43, 44, 45, 46, 47, 48, 49, 50, 51, 52, 53, 54, 55, 56, 57, 58, 59, 60, 61, 62, 63, 64, 65, 66, 67, 79, 80, 81, 82, 83, 84, 85, 86, 87, 88, 89, 90, 91, 97, 98, 111, 112, 113, 114, 115, 116, 117, 118, 119.degradation1, 121, 123, 126, 127, 129, 131, 138, 139, 140, 190.hif_p53_degr, 191, 192, 215, 216, 217, 224, 225
Inv_228	0, 1, 3, 4, 5, 6, 7, 8, 9, 10, 11, 12, 14, 15, 21, 22, 23, 24, 25, 26, 27, 28, 29, 30, 31, 32, 38, 39, 40, 41, 42, 43, 44, 45, 46, 47, 48, 49, 50, 51, 52, 53, 54, 55, 56, 57, 58, 59, 60, 61, 62, 63, 64, 65, 66, 67, 68.absorbed, 69.eating, 70, 71, 72, 73, 74, 75, 76, 77, 78, 79, 82, 83, 97, 98, 113, 114, 115, 116, 117, 118, 119.degradation1, 123, 125, 126, 127, 129, 138, 139, 140, 142, 190.hif_p53_degr, 191, 192, 215, 217, 224, 225, 237
Inv_245	0, 1, 3, 4, 5, 6, 7, 8, 9, 10, 11, 12, 15, 22, 28, 29, 30, 32, 38, 39, 40, 41, 42, 43, 44, 45, 46, 47, 48, 49, 50, 51, 52, 53, 54, 55, 56, 57, 58, 59, 60, 61, 62, 63, 64, 65, 66, 67, 68.absorbed, 69.eating, 70, 71, 72, 73, 74, 75, 76, 77, 78, 79, 80, 81, 82, 83, 84, 85, 86, 87, 88, 89, 90, 91, 97, 98, 111, 112, 113, 114, 115, 116, 117, 118, 119.degradation1, 121, 123, 125, 127, 129, 131, 138, 139, 140, 142, 190.hif_p53_degr, 191, 192, 215, 217, 224, 225, 237
Inv_278	0, 1, 3, 4, 5, 6, 7, 8, 9, 10, 11, 12, 14, 21, 23, 24, 25, 26, 27, 31, 32, 33, 34, 37, 51, 52, 53, 54, 55, 56, 57, 58, 59, 60, 61, 62, 63, 64, 65, 66, 67, 79, 80, 81, 82, 83, 84, 85, 86, 87, 88, 89, 90, 91, 92, 93, 97, 98, 111, 112, 113, 115, 116, 117, 118, 119.degradation1, 120, 123, 126, 129, 130, 131, 138, 139, 140, 190.hif_p53_degr, 191, 192, 215, 216, 217, 224, 225
Inv_279	0, 1, 3, 4, 5, 6, 7, 8, 9, 10, 11, 12, 14, 21, 23, 24, 25, 26, 27, 31, 32, 35, 36, 37, 51, 52, 53, 54, 55, 56, 57, 58, 59, 60, 61, 62, 63, 64, 65, 66, 67, 79, 80, 81, 82, 83, 84, 85, 86, 87, 88, 89, 90, 91, 92, 93, 97, 98, 111, 112, 113, 115, 116, 117, 118, 119.degradation1, 120, 123, 126, 129, 130, 131, 138, 139, 140, 190.hif_p53_degr, 191, 192, 215, 216, 217, 224, 225
Inv_280	0, 1, 3, 4, 5, 6, 7, 8, 9, 10, 11, 12, 14, 21, 23, 24, 25, 26, 27, 31, 32, 38, 39, 40, 41, 42, 43, 44, 45, 46, 47, 48, 49, 50, 51, 52, 53, 54, 55, 56, 57, 58, 59, 60, 61, 62, 63, 64, 65, 66, 67, 79, 80, 81, 82, 83, 84, 85, 86, 87, 88, 89, 90, 91, 92, 93, 97, 98, 111, 112, 113, 115, 116, 117, 118, 119.degradation1, 120, 123, 126, 127, 129, 131, 138, 139, 140, 190.hif_p53_degr, 191, 192, 215, 216, 217, 224, 225
Inv_377	0, 1, 3, 4, 5, 6, 7, 8, 9, 10, 11, 12, 14, 21, 23, 24, 25, 26, 27, 31, 32, 33, 34, 37, 51, 52, 53, 54, 55, 56, 57, 58, 59, 60, 61, 62, 63, 64, 65, 66, 67, 79, 80, 81, 82, 83, 84, 85, 86, 87, 88, 89, 90, 97, 98, 111, 112, 113, 115, 116, 117, 118, 119.degradation1, 120, 122, 123, 126, 129, 130, 131, 138, 139, 140, 190.hif_p53_degr, 191, 192, 215, 216, 217, 224, 225
