# Supplementary material for: Design and Analysis of a Petri Net Model of the Von Hippel-Lindau (VHL) Tumor Suppressor Interaction Network
Source: PLoS One. 2014 Jun 2;9(6):e96986. doi: 10.1371/journal.pone.0096986 (PMC4041725; doi:10.1371/journal.pone.0096986)
Supplement: Table S4 — Composition in terms of invariants of the clusters C8, C9, C10, C11. (PDF) [file pone.0096986.s004.pdf]

C8 (72)	Inv_280, Inv_384, Inv_227, Inv_383, Inv_226, Inv_225, Inv_271, Inv_357, Inv_184, Inv_356, Inv_183, Inv_262, Inv_330, Inv_141, Inv_329, Inv_140, Inv_182, Inv_139, Inv_251, Inv_301, Inv_72, Inv_300, Inv_71, Inv_70, Inv_283, Inv_392, Inv_239, Inv_391, Inv_238, Inv_237, Inv_274, Inv_365, Inv_196, Inv_364, Inv_195, Inv_265, Inv_338, Inv_153, Inv_337, Inv_152, Inv_194, Inv_151, Inv_256, Inv_311, Inv_110, Inv_310, Inv_109, Inv_108, Inv_277, Inv_375, Inv_214, Inv_374, Inv_213, Inv_212, Inv_268, Inv_348, Inv_171, Inv_347, Inv_170, Inv_259, Inv_321, Inv_128, Inv_320, Inv_127, Inv_169, Inv_126, Inv_248, Inv_292, Inv_59, Inv_291, Inv_58, Inv_57
C9 (144)	Inv_381, Inv_223, Inv_380, Inv_222, Inv_279, Inv_278, Inv_378, Inv_219, Inv_377, Inv_218, Inv_221, Inv_217, Inv_270, Inv_269, Inv_261, Inv_260, Inv_354, Inv_180, Inv_353, Inv_179, Inv_351, Inv_176, Inv_350, Inv_175, Inv_327, Inv_137, Inv_326, Inv_136, Inv_324, Inv_133, Inv_323, Inv_132, Inv_178, Inv_174, Inv_135, Inv_131, Inv_298, Inv_68, Inv_297, Inv_67, Inv_250, Inv_249, Inv_295, Inv_64, Inv_294, Inv_63, Inv_66, Inv_62, Inv_390, Inv_236, Inv_389, Inv_235, Inv_282, Inv_281, Inv_387, Inv_232, Inv_386, Inv_231, Inv_234, Inv_230, Inv_273, Inv_272, Inv_264, Inv_263, Inv_363, Inv_193, Inv_362, Inv_192, Inv_360, Inv_189, Inv_359, Inv_188, Inv_336, Inv_150, Inv_335, Inv_149, Inv_333, Inv_146, Inv_332, Inv_145, Inv_191, Inv_187, Inv_148, Inv_144, Inv_309, Inv_107, Inv_308, Inv_106, Inv_255, Inv_254, Inv_306, Inv_103, Inv_305, Inv_102, Inv_105, Inv_101, Inv_372, Inv_210, Inv_371, Inv_209, Inv_276, Inv_275, Inv_369, Inv_206, Inv_368, Inv_205, Inv_208, Inv_204, Inv_267, Inv_266, Inv_258, Inv_257, Inv_345, Inv_167, Inv_344, Inv_166, Inv_342, Inv_163, Inv_341, Inv_162, Inv_318, Inv_124, Inv_317, Inv_123, Inv_315, Inv_120, Inv_314, Inv_119, Inv_165, Inv_161, Inv_122, Inv_118, Inv_289, Inv_55, Inv_288, Inv_54, Inv_247, Inv_246, Inv_286, Inv_51, Inv_285, Inv_50, Inv_53, Inv_49
C10 (52)	Inv_228, Inv_185, Inv_142, Inv_73, Inv_382, Inv_224, Inv_355, Inv_328, Inv_181, Inv_138, Inv_299, Inv_69, Inv_245, Inv_244, Inv_242, Inv_202, Inv_201, Inv_159, Inv_158, Inv_199, Inv_156, Inv_116, Inv_115, Inv_113, Inv_393, Inv_243, Inv_366, Inv_200, Inv_339, Inv_157, Inv_241, Inv_240, Inv_198, Inv_197, Inv_155, Inv_154, Inv_312, Inv_114, Inv_112, Inv_111, Inv_215, Inv_172, Inv_129, Inv_60, Inv_373, Inv_211, Inv_346, Inv_319, Inv_168, Inv_125, Inv_290, Inv_56
C11 (64)	Inv_388, Inv_385, Inv_361, Inv_358, Inv_334, Inv_331, Inv_94, Inv_93, Inv_233, Inv_229, Inv_81, Inv_80, Inv_92, Inv_91, Inv_90, Inv_89, Inv_190, Inv_186, Inv_147, Inv_143, Inv_79, Inv_78, Inv_77, Inv_76, Inv_307, Inv_304, Inv_88, Inv_87, Inv_104, Inv_100, Inv_75, Inv_74, Inv_379, Inv_376, Inv_220, Inv_216, Inv_352, Inv_349, Inv_325, Inv_322, Inv_177, Inv_173, Inv_134, Inv_130, Inv_296, Inv_293, Inv_65, Inv_61, Inv_370, Inv_367, Inv_207, Inv_203, Inv_343, Inv_340, Inv_316, Inv_313, Inv_164, Inv_160, Inv_121, Inv_117, Inv_287, Inv_284, Inv_52, Inv_48
